# Mid-term outcomes of off-pump versus on-pump coronary artery bypass graft surgery; statistical challenges in comparison

**DOI:** 10.1186/s12872-021-02213-0

**Published:** 2021-08-28

**Authors:** Ali Sheikhy, Aida Fallahzadeh, Saeed Sadeghian, Khalil Forouzannia, Jamshid Bagheri, Abbas Salehi-Omran, Masih Tajdini, Arash Jalali, Mina Pashang, Kaveh Hosseini

**Affiliations:** 1 Research Department, Tehran Heart Center, Tehran University of Medical Sciences, Tehran, Iran; 2 Tehran Heart Center, Tehran University of Medical Sciences, North Karegar Ave, P.O. Box: 1411713138, Tehran, Iran

**Keywords:** Coronary artery bypass grafting, Coronary artery bypass, Off-pump, Surgery, Surgical revascularization

## Abstract

**Background:**

Despite several studies comparing off- and on-pump coronary artery bypass grafting (CABG), the effectiveness and outcomes of off-pump CABG still remain uncertain.

**Methods:**

In this registry-based study, we assessed 8163 patients who underwent isolated CABG between 2014 and 2016. Propensity score matching (PSM), inverse probability of weighting (IPW) and covariate adjustment were performed to correct for and minimize selection bias.

**Results:**

The overall mean age of the patients was 62 years, and 25.7% were women. Patients who underwent off-pump CABG had shorter length of hospitalization (*p* < 0.001), intubation time (*p* = 0.003) and length of ICU admission (*p* < 0.001). Off-pump CABG was associated with higher risk of 30-days mortality (OR: 1.7; 95% CI 1.09–2.65; *p* = 0.019) in unadjusted analysis. After covariate adjustment and matching (PSM and IPW), this difference was not statistically significant. After an average of 36.1 months follow-up, risk of MACCE and all-cause mortality didn’t have significant differences in both surgical methods by adjusting with IPW (HR: 1.03; 95% CI 0.87–1.24; *p* = 0.714; HR: 0.91; 95% CI 0.73–1.14; *p* = 578, respectively).

**Conclusion:**

Off-pump and on-pump techniques have similar 30-day mortality (adjusted, PSM and IPW). Off-pump surgery is probably more cost-effective in short term; however, mid-term survival and MACCE trends in both surgical methods are comparable.

**Supplementary Information:**

The online version contains supplementary material available at 10.1186/s12872-021-02213-0.

## Background

Coronary artery bypass grafting (CABG) is one of the most frequently performed surgical procedures worldwide [1] and can be performed in two basic ways: on pump CABG and off pump CABG. CABG is most commonly performed using cardiopulmonary bypass (“on-pump”), which provides prompt cardiac arrest and enables coronary artery anastomosis to be performed on the steady and bloodless field [2]. However, on-pump CABG is associated with the whole-body inflammatory response to the cardiopulmonary bypass, leading to systemic inflammatory response syndrome [3] and postoperative complications, including neurocognitive dysfunction, renal impairment, myocardial depression, and bleeding [4–6]. Consequently, performing CABG on the beating heart without cardiopulmonary bypass (“off-pump”) was first introduced in the mid-1980s to reduce postoperative complications, especially in high-risk patients [7]. Although the long-term effectiveness of off-pump CABG (OPCABG) remains controversial, studies have shown that OPCABG reduces the operation time, the duration of intensive care unit (ICU) admission, the rate of blood transfusion, and early morbidity [4, 8].

Several precious randomized clinical trials (RCTs), cohorts and registry-based studies have compared outcomes for OPCABG and on-pump CABG [9, 10]. However, there are too many controversies in results mainly explained by several pitfalls such as lack of stratification and applying only covariate adjustment and/or propensity score matching (PSM) [11–14].

In the present study, we compared different statistical methods (unadjusted, adjusted and propensity score matching and inverse probability of weighting) to have better comparison in 30-day and mid-term (3 years) results of OPCABG versus on-pump CABG.

## Methods

### Study design

This is a registry-based prospective data analysis study conducted in Tehran Heart Center (THC) [15] clinical registry, which includes patients with coronary artery disease who underwent on-pump or OPCABG between 2014 and 2016. The study approved by Tehran Heart Center ethical board (IR-THC-13799). Therapeutic strategies are based on official guidelines, accordingly none of the patients underwent trial intervention. This study didn’t meet criteria for informed consent whereas patients name kept anonymous except for corresponding author and data base chief, thus “informed consent waiver” obtained from the Tehran Heart Center ethical board. Involving human data was in accordance with guidelines of Declaration of Helsinki.

### Study population

We assessed all patients underwent isolated CABG, also patients with incomplete data were excluded from the study. This left a total of 8163 patients, 1589 of whom underwent OPCABG and 6574 underwent on-pump CABG. After performing 1:1 PS matching, 1312 patients remained in each group.

### Follow-up protocol

The patients were followed at 4 or 6 and 12 months after surgery and yearly thereafter through direct visits. Those who were unable to attend direct clinic visits were followed through telephone interviews. The patients’ demographic characteristics, CAD risk factors (i.e., diabetes mellitus (DM), hypertension (HTN), dyslipidemia, family history of CAD, cigarette smoking (CS), opium consumption, and obesity), laboratory results (hemoglobin and creatinine), history of previous disease (COPD, renal failure and cerebrovascular accident), ejection fraction, number of grafts, and occurrence of major adverse cardio-cerebrovascular events (MACCEs) were recorded.

Diabetes mellitus was defined as fasting plasma glucose ≥ 126 mg/dL and/or random plasma glucose ≥ 200 mg/dL and/or hemoglobin A1c (HbA1c) ≥ 6.5% [16] and/or treatment with either oral hypoglycemic agents or insulin. Hypertension was defined as a minimum systolic blood pressure of 140 mm Hg or a minimum diastolic blood pressure of 90 mm Hg or a history of antihypertensive therapy. Dyslipidemia was defined as the presence of a minimum total cholesterol level of 240 mg/dL, a minimum triglyceride level of 200 mg/dL, or a high-density lipoprotein cholesterol level of less than 40 mg/dL in men and less than 50 mg/dL in women or a minimum low-density lipoprotein cholesterol level of 160 mg/dL, or a history of prescribed lipid medications based on the National Cholesterol Education Program (NCEP) Adult Treatment Plan (ATP) III [17]. A family history of CAD was defined as having a first-degree relative with a history of CAD including acute myocardial infarction or documented CAD (through invasive coronary angiography or computed tomography coronary angiography). Cigarette smoking status was defined as current smoker and determined from the patient’s self-reported status. Opium consumption was defined as the current consumption of opium either orally or through inhalation. Obesity was defined as having a body mass index ≥ 30 kg/m^2^, based on the height and weight recorded prior to the surgery [18].

### Study endpoints

The primary endpoints of this study were in hospital mortality, mid-term mortality and occurrence of mid-term MACCEs (composite of all-cause mortality, acute coronary syndrome, stroke or transient ischemic attack, and the need for repeat revascularization (percutaneous coronary intervention or redo-CABG). Secondary outcomes included length of hospital stay, length of ICU admission and intubation time.

### Surgeon criteria

There were no criteria for surgeon selection, but all surgeons were experienced and they have been conducted at least 200 OPCABG and 400 ONCABG before the study set up.

### Statistical analysis

Normally and skewed distributed continuous variables were presented as mean with standard deviation (SD) and median with 25th and 75th percentiles (interquartile range [IQR] boundaries), respectively. The normality of the variables was assessed using histogram charts as well as central tendency and dispersion measures. They were compared between off- and on- pump groups using student’s *t* test for normally distributed and Mann–Whitney U-test for skewed distributed variables. Categorical variables were expressed as frequency and percentage and were compared between the two abovementioned groups applying the chi-square test.

The adjusted and unadjusted effect of off-pump surgery on 30-days mortality was evaluated using the logistic regression model. The adjusted and unadjusted effects of off-pump surgery on all-cause mortality and MACCE were obtained using Cox’s proportional hazards (PH) model. All adjustments were on detected potential confounders, which affected all outcomes mentioned above in univariate analyses. The standardized mean difference (SMD) reported in Table 2, which used as balance metric to evaluate the difference between distributions of a pre-treatment variable.

The effect of off-pump surgery on 30-day mortality, all-cause mortality and MACCE were also obtained through a stabilized inverse probability weights (IPW) method. Weights were calculated from propensity scores (PS) derived from predicted probabilities of logistic regression of off- vs. on-pump surgery on identified potential confounders. Variable which considered for propensity score matching is listed in Additional file 1: Table S1.

Moreover, we conducted a one-to-one nearest neighborhood propensity score matching (PSM) technique (considering caliper as 0.01) without replacement to compute the effect of off-pump surgery on the abovementioned outcomes (Additional file 1, Table S1).

All results of the methods mentioned above were reported as odds ratios (OR) for 30-days mortality and hazards ratios (HR) for all-cause mortality and MACCE with corresponding 95% confidence intervals (CI).

All statistical analyses were conducted applying IBM SPSS Statistics for Windows, version 22.0 (Armonk, NY: IBM Corp.) and Stata Statistical Software, release 14 (College Station, TX: StataCorp LP).

## Results

### Study population

We assessed all patients with isolated CABG procedure between January 1, 2014 and December 31, 2016. After applying exclusion criteria; including complete loss to follow up (78 patients) and incomplete data registry (332 patients), 6574 patients who underwent on-pump CABG and 1589 patients who underwent OPCABG were included in the analysis. The median length of follow-up was 36.1 months (35.95–36.19 months).

The demographic and preoperative characteristics at the baseline of the unadjusted and PS adjusted populations are shown in Table 1. In brief, patients were 25.7% female and 74.3% male, and the mean age of the patients was 62 years (62.73 years in females and 61.34 years in males).

**Table 1:** Unadjusted and adjusted baseline patients’ characteristics

Baseline characteristics of patients before PS matching					Baseline characteristics of patients after PS matching		
	Total (n = 8163)	Off-pump (n = 1589)	On-pump (n = 6574)	*p* value	Off-pump (m = 1312)	On-pump (m = 1312)	*p* value
Female	25.7% (2096)	26.4% (420)	25.5% (1676)	0.462	26.2% (351)	25.8% (346)	0.860
Age	62 ± 10	62 ± 10	62 ± 9	0.260	62.03 ± 9.88	62.00 ± 9.41	0.964
BMI							
< 30	75.1% (6096)	74.3% (1170)	75.3% (4926)	0.423	74.9% (331)	74.1 (992)	0.658
≥ 30	24.9% (2016)	25.7% (404)	24.7% (1612)		25,1% (336)	25.9% (347)	
Hb	13.89 ± 1.74	13.76 ± 1.79	13.92 ± 1.72	0.002	13.92 ± 1.71	13.85 ± 1.76	0.57
Graft number*	3 [3, 4]	3 [2, 3]	3 [3, 4]	< 0.001	3 [2, 3]	3 [2, 3]	0.942
Creatinine*	0.90 [0.77,1.07]	0.90 [0.77,1.08]	0.97 [0.77,1.06]	0.108	0.90 [0.77, 1.07]	0.90 [0.77, 1.09]	0.739
eGFR	86.38 [67.22, 107.74]	85.5 [65.68, 107.89]	86.44 [67.78, 107.64]	0.159	85.28 [65.71, 106.82]	85.83 [66.7, 107.65]	0.388
Diabetes	41.2% (3363)	43% (681)	40.8% (2682)	0.122	42.6% (570)	44.7% (598)	0.293
Hypertension	58.4% (4759)	57.1% (903)	58.7% (3586)	0.266	57.4% (769)	60.5% (810)	0.116
Dyslipidemia	53.8% (4386)	55.1% (870)	53.5% (3516)	0.280	55.9% (748)	56.5% (756)	0.758
Positive family history	30.0% (2449)	27.7% (440)	30.6% (2009)	0.029	29.4% (393)	29.4% (394)	< 0.999
Opium	18.6% (1509)	16.6% (260)	19.1% (1249)	0.026	17.2% (230)	17.7% (237)	0.760
Current cigarette smoking	20.4% (1656)	18.0% (283)	21.0% (1373)	0.01	18.7% (251)	18.9% (253)	0.961
Ejection fraction							
≥ 50	44.9% (3612)	41.1% (637)	45.8% (2975)	< 0.001	42.2% (565)	43.2% (579)	0.612
< 50	55.1% (4429)	58.9% (911)	54.2% (3518)		57.8% (774)	56.8% (760)	
Left main stenosis	11.2% (913)	11.7% (186)	11.1% (727)	0.490	11.3% (151)	11.5% (154)	0.903
Pre surgery PCI	10.2% (834)	12.5% (199)	9.7% (635)	< 0.001	11.8% (158)	12.2% (164)	0.766
Renal failure	2.8% (225)	4.0% (63)	2.5% (162)	< 0.001	3.4% (45)	2.3% (30)	0.103
COPD	3.7% (302)	4.6% (71)	3.5% (231)	0.065	4.0% (53)	3.7% (49)	0.762
Cerebrovascular accident	10.1% (820)	10.4% (163)	10.0% (657)	0.14	10.2% (137)	11.4% (153)	0.351
Urgent operation	1.2% (98)	0.9% (14)	1.3% (84)	0.671	0.7% (9)	1.1% (15)	0.305
Previous myocardial infarction							
No history	70.2% (5730)	73.1% (1161)	69.5% (4569)	0.001	72.0% (964)	75.7% (1014)	0.148
≤ 7 days	10.4% (848)	10.1% (160)	10.5% (686)		10.2% (136)	9.3% (125)	
8–21 days	7.1% (582)	5.0% (79)	7.7% (503)		5.3% (71)	4.7% (63)	
> 21 days	12.3% (1003)	11.9% (189)	12.4% (814)		12.5% (168)	10.2% (137)	

*P value < 0.05 considered as significant

*BMI* body mass index, *Hb* hemoglobin, *eGFR* estimated glomerular filtration rate, *PCI* percutaneous coronary intervention, *COPD* chronic obstructive pulmonary disease

After performing PS matching the differences in characteristics between two groups were completely even, except for the amount of hemoglobin which was significantly higher in on-pump group in both adjusted and unadjusted patients, but the difference was not clinically significant. Table 2 demonstrates standardized mean differences of each variable to asses balance checking for each adjustment method.

**Table 2:** Standardized mean differences (SMD) percentage of characteristic variables

	Unadjusted	PSM	IPW
eGFR	0.04	0.012	0.042
Age	0.045	0.015	0.055
Hb	0.094	0.037	0.062
Gender	0.015	0.012	0.015
Dyslipidemia	0.027	0.015	0.036
Diabetes	0.045	0.029	0.039
Hypertension	0.02	0.024	0.005
Positive family history	0.049	0.008	0.036
Opium	0.067	0.039	0.044
Current cigarette smoker	0.077	0.002	0.053
EF	0.097	0.003	0.059
Left main stenosis	0.007	0.005	0.012
Pre surgery PCI	0.107	0.054	0.045
BMI	0.014	0.002	0.006
Urgent operation	0.049	0.008	0.034
COPD	0.046	0.012	0.006
Cerebrovascular accident	0.018	0.052	0.027
Previous myocardial infarction (≤ 7 days)	0.109	0.01	0.012
Previous myocardial infarction (8–21 days)	0.122	0.043	0.075
Previous myocardial infarction (> 21 days)	0.011	0.016	0
Graft number	0.907	0.001	0.589

### Primary outcomes

#### 30-days mortality

OPCABG was associated with higher risk of 30-days mortality (OR: 1.7; 95% CI 1.09–2.65; *p* = 0.019) in unadjusted analysis. After covariate adjustment and matching (PSM and IPW), this difference was not statistically significant. However, based on PSM and IPW the trend was in favor of on-pump CABG (lower 30-days mortality) (OR 2.02; 95% CI 0.94–4.35; *p* = 0.073; OR: 1.51; 95% CI 0.93–2.45; *p* = 0.092, respectively).

#### Mid-term MACCE

OPCABG was also associated with higher risk of MACCE at 3 years than on-pump CABG (HR: 1.26; 95% CI 1.1–1.44; *p* = 0.001), hence; the effect was reduced after using PSM (HR: 1.19; 95% CI 0.97–1.46; *p* = 0.089), also there were no significant differences between ONCABG and OPCABG in IPW modeling (HR: 1.03; 95% CI 0.87–1.24; *p* = 0.714).

#### Mid-term all-cause mortality

Moreover, OPCABG was associated with higher risk of all-cause mortality (HR: 1.33; 95% CI 1.12–1.58; *p* = 0.001) and the effect was not significant after using PSM and IPW adjustment method (HR: 1.08; 95% CI 0.83–1.41; *p* = 0.432; HR: 0.91; 95% CI 0.73–1.14; *p* = 578, respectively).

**Fig. 1:**
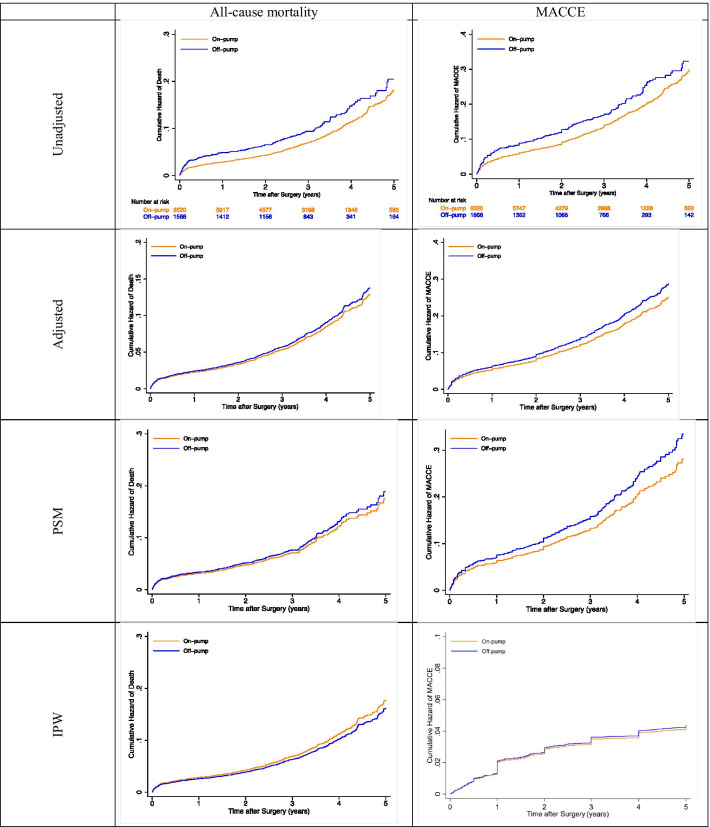
Crude and adjusted cumulative Hazard of death and MACCE at 5 years after surgery

None of the primary outcomes were significantly higher in OPCABG after applying covariate adjustment (Table 3, Fig. 1).

**Table 3:** Effect of off-pump versus on-pump surgery on 30-days mortality, all-cause mortality, and MACCE

	First 30 days mortality		MACCE		All-cause mortality	
	OR (CI 95%)	*p* value	HR (CI 95%)	*p* value	HR (CI 95%)	*p* value
Unadjusted	1.70 [1.09, 2.65]	0.019	1.26 [1.10, 1.44]	0.001	1.33 [1.12, 1.58]	0.001
Adjusted	1.42 [0.89, 2.27]	0.141	1.15 [0.98, 1.34]	0.087	0.97 [0.87, 1.21]	0.522
PSM	2.02 [0.94, 4.35]	0.073	1.19 [0.97, 1.46]	0.089	1.08 [0.83, 1.41]	0.432
IPW	1.51 [0.93, 2.45]	0.092	1.03 [0.87, 1.24]	0.714	0.91 [0.73, 1.14]	0.578

### Secondary outcomes

As shown in Table 4, length of hospitalization, intubation time and length of ICU admission were significantly longer in patients with on-pump CABG procedure (mean: 7.8 vs 6.6 days; *p* < 0.001; 14.3 vs 12.0 h; *p* = 0.003; 46.3 vs 32.8 h; *p* < 0.001, respectively).

**Table 4:** Secondary outcomes compared in off- vs on-pump CABG

	On-pump	Off-pump	*p* value
Length of hospitalization (day)	7.896 ± 5.292	6.687 ± 3.569	< 0.001
Intubation time (hour)	14.354 ± 39.968	12.092 ± 23.548	0.003
Length of ICU admission (hour)	46.351 ± 84.146	32.812 ± 35.249	< 0.001

## Discussion

The intents of the present study were to compare benefits and detriments of off-pump and on-pump CABG procedure. We also compared four statistical analysis methods (unadjusted, adjusted, PSM and IPW). Based on this large observational registry-based study, short term (30 days) mortality rate was not different between two types of procedures, but according to the reduction in duration of hospital stays, ICU admission, and intubation time, OPCABG is probably more cost-effective. In the terms of mid-term (3 years) mortality and MACCE, this study showed that both surgical strategies have same mid-term outcomes.

### Statistical challenge

An obvious disadvantage of an observational cohort study is the risk of selection bias, therefore in this study, we used different methods to minimize the impact of such bias in the final analysis. First, we implemented the more conventional method, covariate adjustment, to control for covariate effects. Second, we used propensity score matching (PSM), which provides excellent covariate balance but its main disadvantage is exclusion of unmatched patients from the analysis. Finally, to overcome this disadvantage of PSM method, we implemented inverse probability weighting (IPW), which retains data from all study population and provides perfect covariate balance. In other words, IPW is easy to implement, moreover, it uses the whole data set and by reweighting individuals, increases the weight of those with unexpected exposures, eventually IPW creates a pseudo-population in which the covariates are balanced excellent between treatment groups. Here, we showed that the higher slightly significant risk of MACCE in OPCABG became unsignificant only after applying IPW; this may emphasize the importance of applying IPW in such non randomized and large sample size studies. To the best of our knowledge, this is one the few studies [19] that applied and compared all these four methods to assess short and mid-term outcomes of off-pump and on-pump CABG.

Short-term mortality is mostly due to heart failure, hemorrhage, dysrhythmia, and respiratory failure [20]. In agreement to our results, some remarkable meta-analysis studies showed there are no significant differences in selecting each procedure [21, 22]. Likewise, in high-risk patients (defined as: > 54 years of age, had a EuroSCORE ≥ 5, and 3-vessel disease) there were no differences in short-term mortality [23]. Besides, some valuable clinical trials such as CORONARY and GOPCABE [4, 24–26] showed that mortality rate have significant differences between off-pump and on-pump CABG. Keeling et al. [27] conducted a study among patients with an ejection fraction of less than 30%. According to their results, OPCABG had better short-term results compared with on-pump CABG; however, the long-term mortality was not evaluated in their study.

Our study showed that mid-term MACCE and mortality (based on PSM and IPW) is similar in OPCABG and ONCABG.

Some large scaled clinical trials presented equivalent mid- and long-term mortality in off-pump and on-pump CABG [9, 10, 28]. In all of these studies odds ratio was above 1 but not significant; hence, a convincing meta-analysis study by Thakur et al. [21], using mentioned clinical trials [9, 10, 28], showed that off-pump CABG causes higher mortality rate (about 18%) in long-term. This controversy is maybe due to effect of small sample size in clinical trial studies.

GOPCABE trial [29], German Off-Pump Coronary Artery Bypass Grafting in Elderly Patients (more than 75 years), revealed that there were no differences in term of 5-years mortality; hence, incomplete revascularization was 5% higher in off-pump strategy. Therefore, on-pump CABG may be more beneficial in elderly patients.

In addition, large scaled observational studies were in contrast to our results. Chikwee et al. [13] evaluated 10-years mortality in 22,245 patients who underwent off- and on-pump CABG, they established higher mortality rate (about 11%) in OPCABG group. Another study conducted by Hu et al. [30], showed that off-pump CABG was associated with increased long-term risks of repeat revascularization and major vascular events. Hannan et al. [14], showed that off-pump CABG was associated with lower in-hospital mortality than on-pump CABG, but long-term outcomes were similar within two groups. Williams et al. [31] also, showed that OPCABG was associated with higher risk for revascularization during the follow-up (2.6 years). A meta-analysis conducted by Gaudino et al. [32] found that OPCABG was associated with higher risk of late mortality and late repeated revascularization, especially when the follow-up duration was > 3 years.

The most important *strength* of our study was using four types of analysis; unadjusted, covariate adjustment, PSM, and IPW to minimize the selection bias in large sample size data registry. We adjusted the results and matched the groups with many known confounding predictors of MACCE and mortality which is essential to have better comparative conclusion.

Our study had some *limitations* that should be considered to interpret the findings. Our results may be affected by unmeasured variables such as intraoperative bypass graft assessment, surgeon expertise, surgical techniques and post-operative variables that may affect the outcome. However, in our study 99.4% of off-pump surgeries were done by Dr. K.F and thus surgeon expertise and surgical techniques are almost equal within off-pump CABG group.

Follow up period may also affect the results. Our results were based on median 3 years follow-up which may differ in longer follow up periods. In addition, results of single-center studies may not be applicable in general. However, THC is the referral educational university (under the authority of Tehran University of Medical Sciences) which serve patients from all parts of the country.

The primary endpoints of present study were MACCE and mortality, however, to have better comparison between surgical techniques, we should also consider other post-operative complications such as: surgical site infection, sepsis, post-operative renal failure, anemia (blood transfusion) and other events.

In conclusion off-pump and on-pump techniques have similar 30-day mortality (adjusted, PSM and IPW) and are probably more cost-effective in short term, also mid-term trends in both MACCE and mortality are equal in both surgical methods. Further large sample size randomized studies should preform to compare the pure and unbiased results of these two techniques.

## Supplementary Information

**Additional file 1:Table S1.** Variables used in propensity score matching and weighting. **Codes S1.** Used codes in STATA for propensity score measurement and matching. **Figure S1.** PS distribution and overlapping of unmatched and matched population.

## Data Availability

The data that support the findings of this study are available on request from the corresponding author (K.H).
