# Supplementary material for: Mid-term outcomes of off-pump versus on-pump coronary artery bypass graft surgery; statistical challenges in comparison
Source: BMC Cardiovasc Disord. 2021 Aug 28;21:412. doi: 10.1186/s12872-021-02213-0 (PMC8403445; doi:10.1186/s12872-021-02213-0)
Supplement: Supplementary file 1 — Additional file 1:Table S1. Variables used in propensity score matching and weighting. Codes S1. Used codes in STATA for propensity score measurement and matching. Figure S1. PS distribution and overlapping of unmatched and matched population. [file 12872_2021_2213_MOESM1_ESM.docx]

| Table S1  **Variables used in propensity score matching and weighting** |
| --- |
| **Demographic**  Female  Age  BMI <30 and ≥30  Opium |
| **Medical history**  Diabetes  Hypertension  Dyslipidemia  COPD  Cerebrovascular accident  Pre surgery PCI  Positive family history |
| **Preoperative lab test**  GFR  Hb |
| **Cardiac status**  Graft number  Ejection fraction ≥50 and **<50**  LM stenosis  Previous myocardial infarction (No history, ≤7 days, 8-21 days, **>**21days)  Current cigarette smoking |
| **Surgical decision**  Urgent operation |

Used codes in STATA

IPW:

logistic Off_PumpSurgery_new

predict pn_p2d

logistic Off_PumpSurgery_new eGFR_cat Age Hb i.Gender i.Dyslipidemia i.Diabetes i.Hypertansion i.PositiveFamilyHx i.Opium_code i.currentCS i.EF_Cat_2 i.LM i.Pre_Surg_PCI i.BMI_CODE i.StatusofProcedure i.COPD i.CerebrovascularAccident i.Pre_CABG_MI_Interval Graft_num

predict pd_p2d

gen wt_p2d=.

replace wt_p2d=pn_p2d/pd_p2d if Off_PumpSurgery_new==1

replace wt_p2d=(1-pn_p2d)/(1-pd_p2d) if Off_PumpSurgery_new==0

PSM:

psmatch2 Off_PumpSurgery_new eGFR_cat Age Hb i.Gender i.Dyslipidemia i.Diabetes i.Hypertansion i.PositiveFamilyHx i.Opium_code i.currentCS i.EF_Cat_2 i.LM i.Pre_Surg_PCI i.BMI_CODE i.StatusofProcedure i.COPD i.CerebrovascularAccident i.Pre_CABG_MI_Interval Graft_num, caliper(0.01) noreplace logit odds neighbor(1)


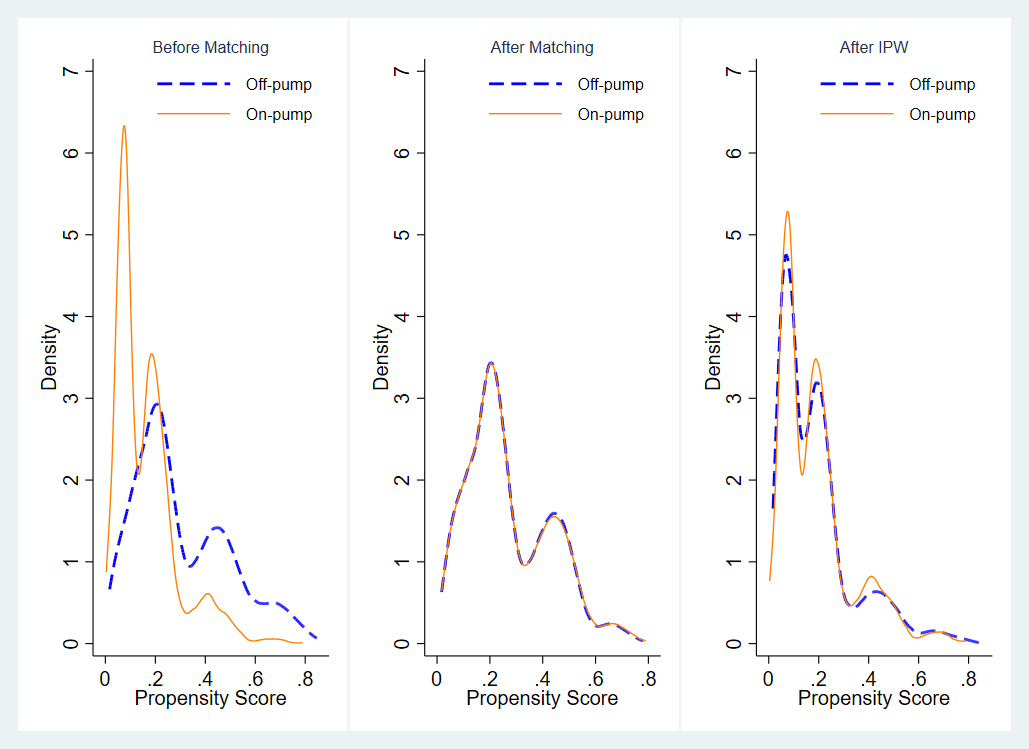


Figure S1. PS distribution and overlapping of unmatched and matched population
